# Economic Analysis of Tissue-First, Plasma-First, and Complementary NGS Approaches for Treatment-Naïve Metastatic Lung Adenocarcinoma

**DOI:** 10.3389/fonc.2022.873111

**Published:** 2022-05-20

**Authors:** Szu-Chun Yang, Chien-Chung Lin, Yi-Lin Chen, Wu-Chou Su

**Affiliations:** ^1^ Department of Internal Medicine, National Cheng Kung University Hospital, College of Medicine, National Cheng Kung University, Tainan, Taiwan; ^2^ Molecular Diagnosis Laboratory, Department of Pathology, National Cheng Kung University Hospital, Tainan, Taiwan; ^3^ Department of Medical Laboratory Science and Biotechnology, College of Medicine, National Cheng Kung University, Tainan, Taiwan; ^4^ Department of Oncology, National Cheng Kung University Hospital, College of Medicine, National Cheng Kung University, Tainan, Taiwan

**Keywords:** liquid biopsy, ctDNA, next-generation sequencing, cost analysis, cost minimization

## Abstract

**Background:**

To compare the testing costs and testing turnaround times of tissue-first, plasma-first, and complementary next-generation sequencing (NGS) approaches in patients with treatment-naïve metastatic lung adenocarcinoma.

**Materials and Methods:**

We developed a decision tree model to compare three different approaches. Patients were entered into the model upon cancer diagnosis and those with both insufficient tissue specimens and negative liquid-based NGS were subjected to tissue re-biopsy. Actionable gene alterations with the U.S. Food and Drug Administration (FDA)-approved therapies included epidermal growth factor receptor (*EGFR*) mutation, anaplastic lymphoma kinase (*ALK*) gene rearrangement, *ROS* proto-oncogene 1 (*ROS1*) rearrangement, *B-Raf* proto-oncogene (*BRAF*) *V600E* mutation, rearranged during transfection (*RET*) gene rearrangement, mesenchymal-epithelial transition factor (*MET*) mutation, neurotrophic tyrosine receptor kinase (*NTRK*) gene rearrangement, *K-Ras* proto-oncogene (*KRAS*) *G12C* mutation, and human epidermal growth factor receptor 2 (*HER2*) mutation. Model outcomes were testing costs, testing turnaround times, and monetary losses taking both cost and time into consideration. We presented base-case results using probabilistic analysis. Stacked one-way and three-way sensitivity analyses were also performed.

**Results:**

In terms of testing costs, tissue-first approach incurred US$2,354($1,963–$2,779) and was the most cost-efficient strategy. Complementary approach testing turnaround time (days) of 12.7 (10.8 to 14.9) was found as the least time-consuming strategy. Tissue-first, complementary, and plasma-first approaches resulted in monetary losses in USD of $4,745 ($4,010–$5,480), $6,778 ($5,923–$7,600), and $7,006 ($6,047–$7,964) respectively, and identified the same percentage of patients with appropriate FDA-approved therapies. Costs for liquid-based NGS, EGFR mutation rates, and quantity of tissue specimens were the major determinants in minimizing monetary loss. Plasma-first approach would be the preferable strategy if its testing price was reduced in USD to $818, $1,343, and $1,869 for populations with *EGFR* mutation rates of 30%, 45%, and 60% respectively.

**Conclusion:**

The tissue-first approach is currently the best strategy in minimizing monetary loss. The complementary approach is an alternative for populations with a low *EGFR* mutation rate. The plasma-first approach becomes increasingly preferable as *EGFR* mutation rates gradually increase.

## Introduction

Targeted therapies have changed the landscape of lung cancer treatments. Administering targeted therapies to patients with metastatic lung adenocarcinoma harboring actionable gene alterations improves tumor response and survival outcomes. Timely identification of these actionable gene alterations can facilitate early initiation of appropriate therapies (1). Tissue-based next-generation sequencing (NGS) tests all actionable gene alterations and has been at the forefront in guiding appropriate therapies (2). An inherent disadvantage of tissue-based NGS is that an insufficient quantity of tissue specimens requires an invasive re-biopsy. Liquid-based NGS is minimally invasive and has a rapid turnaround time. According to the new consensus statement from the International Association for the Study of Lung Cancer, liquid-based NGS is emerging as the initial (“plasma-first”) approach or “complementary” to tissue-based NGS at the time of diagnosis (3).

A major drawback of plasma-first NGS approach is that the high probability of false-negative results can require additional tissue analysis which is time-consuming. Complementary NGS approach, with concurrent examinations of tissue- and plasma-based NGS, is a time-saving strategy; however, a caveat that cannot be neglected is its high price (4). Previous research has shown that the prevailing tissue-based NGS is more cost-efficient and less time-consuming when compared with sequential or exclusionary single-gene testing (5). Cost evaluation studies using Italian multicenter data also highlighted that the adoption of NGS saves personnel time and reduces the overall cost of testing (6, 7). Another study found that in patients with insufficient tissue specimens, liquid-based NGS adds lives with a modest budget impact (8). However, to date, there is no study directly comparing the testing costs and testing turnaround times of tissue-first, plasma-first, and complementary NGS approaches for patients with treatment-naïve metastatic lung adenocarcinoma.

We hypothesized that plasma-first NGS approach is not a cost-efficient and time-saving strategy in comparison with tissue-first and complementary NGS approaches and all three NGS approaches can diagnose actionable gene alterations with similar accuracy. By conducting a decision-tree analysis and addressing re-biopsy and false-negative issues, we aimed to verify our hypothesis.

## Materials and Methods

We conducted the analyses from the U.S. societal perspective. The target population consisted of patients who had newly-diagnosed metastatic lung adenocarcinoma. This model-based analysis was given an exemption from ethical review by the National Cheng Kung University Hospital (A-EX-111-001).

### Model Overview

**Figure 1:**
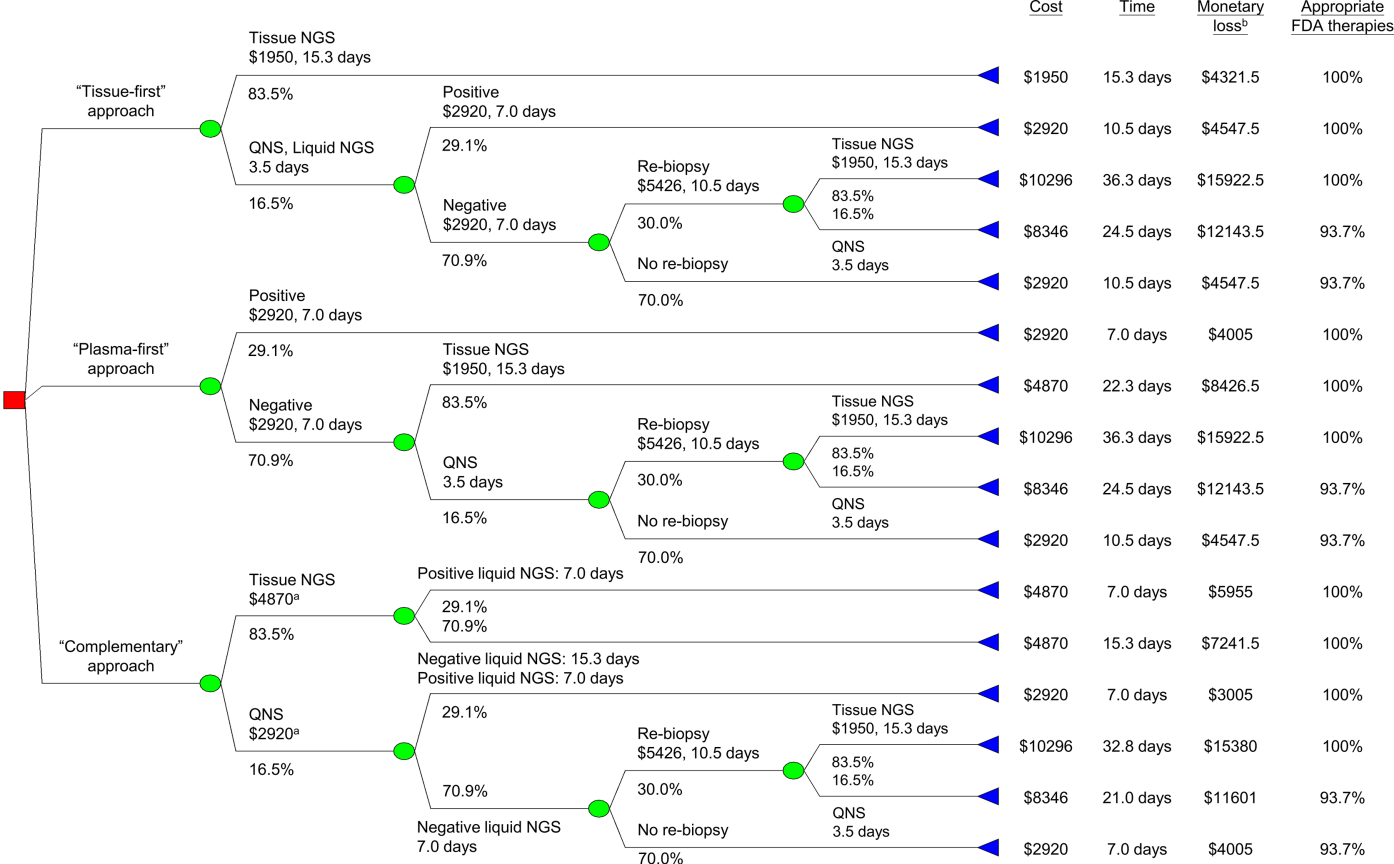
Decision tree analysis for minimizing monetary loss. ^a^Including cost for liquid-based NGS. ^b^Monetary loss included both testing and productivity costs, latter was the product of turnaround time and average wage (**Table 1**). FDA, U.S. Food and Drug Administration. NGS, next-generation sequencing; QNS, quantity not sufficient.

We developed a decision tree model to compare tissue-first, plasma-first, and complementary approaches of NGS testing for treatment-naïve metastatic lung adenocarcinoma. **Figure 1** depicts the model structure showing how patients enter into the model after being diagnosed with lung cancer and tissue samples were available for tumor genotyping. In tissue-first NGS approach, tissue-based NGS was used to test all actionable gene alterations. Tissue specimens with a quantity not sufficient (QNS) for tissue-based NGS were followed by liquid-based NGS and if results were negative, a re-biopsy was considered. In plasma-first NGS approach, patients were initially tested for liquid-based NGS and if results were negative, this was followed by a tissue-based NGS; however, for specimens with a QNS, a tissue re-biopsy was considered. In the complementary NGS approach, both tissue- and liquid-based NGS were simultaneously tested in the beginning. For tissue specimens with sufficient quantity tissue-based NGS, the turnaround time was determined when the results of the liquid-based NGS were made available. For specimens with a QNS for tissue-based NGS and liquid-based NGS with negative results, tissue re-biopsy was considered.

### Model Inputs

Testing costs of the tissue-based NGS and liquid-based NGS were determined on the grounds of the reimbursement rates of Centers for Medicare & Medicaid Services (**Table 1**) (9). The cost of re-biopsy, including both inpatient and outpatient costs, as well as the cost for procedure-related complications, was derived from Medicare claims data from previous research (10, 11). To calculate the average daily wage, we used the mean wage of all occupations from the public database of the Bureau of Labor Statistics (12). For tissue specimens with a QNS for a tissue-based NGS, the pre-analytical time was 3.5 days (1). The turnaround time of the tissue-based NGS was 15.3 days, which included both pre-analytical and in-laboratory time (1). For the liquid-based NGS, the turnaround time was 7 days (13) and the time to re-biopsy results was 10.5 days (5). Because each testing strategy included testing the programmed death-ligand 1 (PD-L1) expression level, we did not consider its additional cost and time when there were no actionable gene alterations.

**Table 1:** Model inputs.

Parameter	Baseline value	Range		Distribution	References for baseline value
		Minimum	Maximum		
Testing cost (US$)					
Tissue-based NGS (CPT: 0022U)	1950	0	3900	Gamma (100,19.50)	(9)
Liquid-based NGS (CPT: 81455)	2920	0	5840	Gamma (100,29.20)	(9)
Re-biopsy	5426	0	10,852	Gamma (100,54.26)	(10, 11)
Average wage (US$/day)	155	0	310	Gamma (100,1.55)	(12)
Turnaround time (day)					
Pre-analytical^a^	3.5	0	7.0	Gamma (100,0.035)	(1)
Tissue-based NGS	15.3	0	30.6	Gamma (100,0.153)	(1)
Liquid-based NGS	7.0	0	14.0	Gamma (100,0.07)	(13)
Re-biopsy	10.5	0	21.0	Gamma (100,0.105)	(5)
Gene alteration rate					
* EGFR*	12.5%	0%	100%	Beta (12.5,87.5)	(14)
* ALK*	4.0%			Beta (4,96)	(14)
* ROS1*	1.0%			Beta (1,99)	(14)
* BRAF V600E*	1.0%			Beta (1,99)	(14)
* RET*	1.5%			Beta (1.5,98.5)	(14)
* MET*	3.5%			Beta (3.5,96.5)	(14, 15)
* NTRK*	0.2%			Beta (1,540)	(16)
* KRAS G12C*	11.7%			Beta (11.7,88.3)	(14)
* HER2*	1.0%			Beta (1,99)	(14)
Re-biopsy input					
Sensitivity of liquid-based NGS^b^	80.0%	0%	100%	Beta (48,12)	(17)
Quantity not sufficient (QNS) for tissue-based NGS	16.5%	0%	100%	Beta (14,71)	(18)
Patients re-biopsied of those in need	30.0%	0%	100%	Beta (30,70)	(5)
Patients with appropriate FDA-approved therapies using tissue-based NGS	100%			–	–
Patients with appropriate FDA-approved therapies using liquid-based NGS^c^	93.7%			Beta (177,12)	(17)

a Pre-analytical time of tissue-based NGS. The turnaround time of tissue-based NGS includes both the pre-analytical and in-laboratory time.

b Negative liquid-based NGS (%) = 100% - (EGFR/ALK/ROS1/BRAF V600E/RET/MET/NTRK/KRAS G12C/HER2 alteration rate (14–16): 36.4% × sensitivity of liquid-based NGS (17): 80%) = 70.9%.

c 100% - false-negative rate (%) of liquid-based NGS.

ALK, anaplastic lymphoma kinase; BRAF, B-Raf proto-oncogene; CPT, Current Procedural Terminology code; EGFR, epidermal growth factor receptor; FDA, U.S. Food and Drug Administration; HER2, human epidermal growth factor receptor 2; MET, mesenchymal-epithelial transition; NGS, next generation sequencing; NTRK, neurotrophic tyrosine receptor kinase; KRAS, K-Ras proto-oncogene; RET, rearranged during transfection; ROS1, ROS proto-oncogene 1.

We obtained the gene alteration rates from previous literature (14–16). Actionable gene alterations for which the United States Food and Drug Administration (FDA) has approved targeted therapies included epidermal growth factor receptor (*EGFR*) mutation, anaplastic lymphoma kinase (*ALK*) gene rearrangement, *ROS* proto-oncogene 1 (*ROS1*) rearrangement, *B-Raf* proto-oncogene (*BRAF*) *V600E* mutation, rearranged during transfection (*RET*) gene rearrangement, mesenchymal-epithelial transition factor (*MET*) mutation, neurotrophic tyrosine receptor kinase (*NTRK*) gene rearrangement, and *K-Ras* proto-oncogene (*KRAS*) *G12C* mutation (2). Because *MET* amplification and human epidermal growth factor receptor 2 (*HER2*) mutation were emerging as potential biomarkers for lung cancer with FDA-approved therapies (19, 20), they were also regarded as actionable gene alterations. To calculate the probability (%) of patients with negative liquid-based NGS, we multiplied the rate of actionable gene alterations (14–16) by the sensitivity of liquid-based NGS (17) and subtracted the product from 100. A total of 16.5% of patients had tissue specimens with a QNS for tissue-based NGS (18). Patients with both insufficient tissue specimens and negative liquid-based NGS were subjected to re-biopsy. Of those requiring re-biopsy, only 30.0% had the procedure done (5). We assumed that the tissue-based NGS identified all actionable gene alterations. For tumor genotyping established *via* negative liquid-based NGS, we considered the false-negative rate of the liquid-based NGS (17) while calculating the percentage of patients with appropriate FDA-approved therapies.

### Model Outcomes

We aim to minimize the testing costs and testing turnaround times. Considering both testing costs and testing turnaround times, we calculated the monetary loss using the following equation:


$$\begin{array}{c} Monetary\;loss\;=\;Testing\;costs\;+\;Time\;costs\\ =Testing\;costs\;+\;Time\;\times \;Average\;wage \end{array}$$

where monetary loss is determined not only by the direct medical costs associated with testing but also by the indirect productivity lost while waiting for test results. Productivity costs were regarded as a lower bound of willingness-to-accept (21). We adjusted the annual medical inflation rates and converted all costs to 2021 U.S. dollars (USD).

Some patients with insufficient tissue specimens and negative liquid-based NGS might not undergo re-biopsy or might experience re-biopsy failure. As a result, actionable gene alterations harbored by these patients might not be detected due to false-negative results of the liquid-based NGS. Therefore, we also compared the percentages of patients with appropriate FDA-approved therapies.

### Sensitivity Analyses

To address the effect of model parameter uncertainty on the outcomes, we conducted a probabilistic analysis using cohort simulation with 1,000 iterations. Distributions of different input parameters are detailed in **Table 1**. Base-case results were presented as mean values and 95% prediction intervals. To test the robustness of our results, we performed stacked one-way sensitivity analysis by varying the input parameters in broad ranges (**Table 1**) and determined the best strategy at each value. We also conducted a three-way sensitivity analysis by varying the costs of liquid-based NGS, the probability of specimens insufficient for tissue-based NGS, and the *EGFR* mutation rate simultaneously. Amua software (version 0.3.0) was used to perform the analysis.

In the base-case analysis, we used a deoxyribonucleic acid (DNA) and ribonucleic acid (RNA) panel. A sensitivity analysis using a DNA panel, which incurred less cost and could not detect most *ALK/ROS1/RET/NTRK* gene rearrangements and *MET exon14* skipping, was performed.

### Scenario Analysis Using Taiwanese Data

Due to a low daily testing volume, the price of the liquid-based NGS in Taiwan has remained high and the gene alteration rates are different from those in the U.S., thus, we applied Taiwanese data (22–30) (**Supplementary Table 1**) to the model for scenario analysis (**Supplementary Figure 1**).

## Results

### Base-Case Results

For a patient with treatment-naïve metastatic lung adenocarcinoma, tissue-first, complementary, and plasma-first NGS approaches resulted in monetary losses in USD of $4,745 (95% prediction interval: $4,010–$5,480), $6,778 ($5,923–$7,600), and $7,006 ($6,047–$7,964), respectively (**Table 2**). In terms of testing costs, the tissue-first NGS approach incurred $2,354 ($1,963–$2,779) and was the most cost-efficient strategy. The testing turnaround time for complementary NGS approach was 12.7 days (10.8 to 14.9 days), being the least time-consuming strategy. Three different NGS approaches identified the same percentage of patients with appropriate FDA-approved therapies.

**Table 2:** Base-case results^a^.

	Cost (US$)	Time (day)	Monetary loss^b^ (US$)	Patients with appropriate FDA-approved therapies
“Tissue-first” NGS approach	2354 (1963 to 2779)	15.3 (12.9 to 18.0)	4745 (4010 to 5480)	99.4% (98.9 to 99.8%)
“Complementary” NGS approach	4795 (4085 to 5453)	12.7 (10.8 to 14.9)	6778 (5923 to 7600)	99.4% (98.9 to 99.8%)
“Plasma-first” NGS approach	4316 (3659 to 4946)	17.2 (14.7 to 20.1)	7006 (6047 to 7964)	99.4% (98.9 to 99.8%)

a Values in parentheses denote the 95% prediction intervals.

b Monetary loss included testing and productivity costs, latter was the product of turnaround time and average wage.

FDA, U.S. Food and Drug Administration; NGS, next generation sequencing.

### Sensitivity Analyses

**Figure 2:**
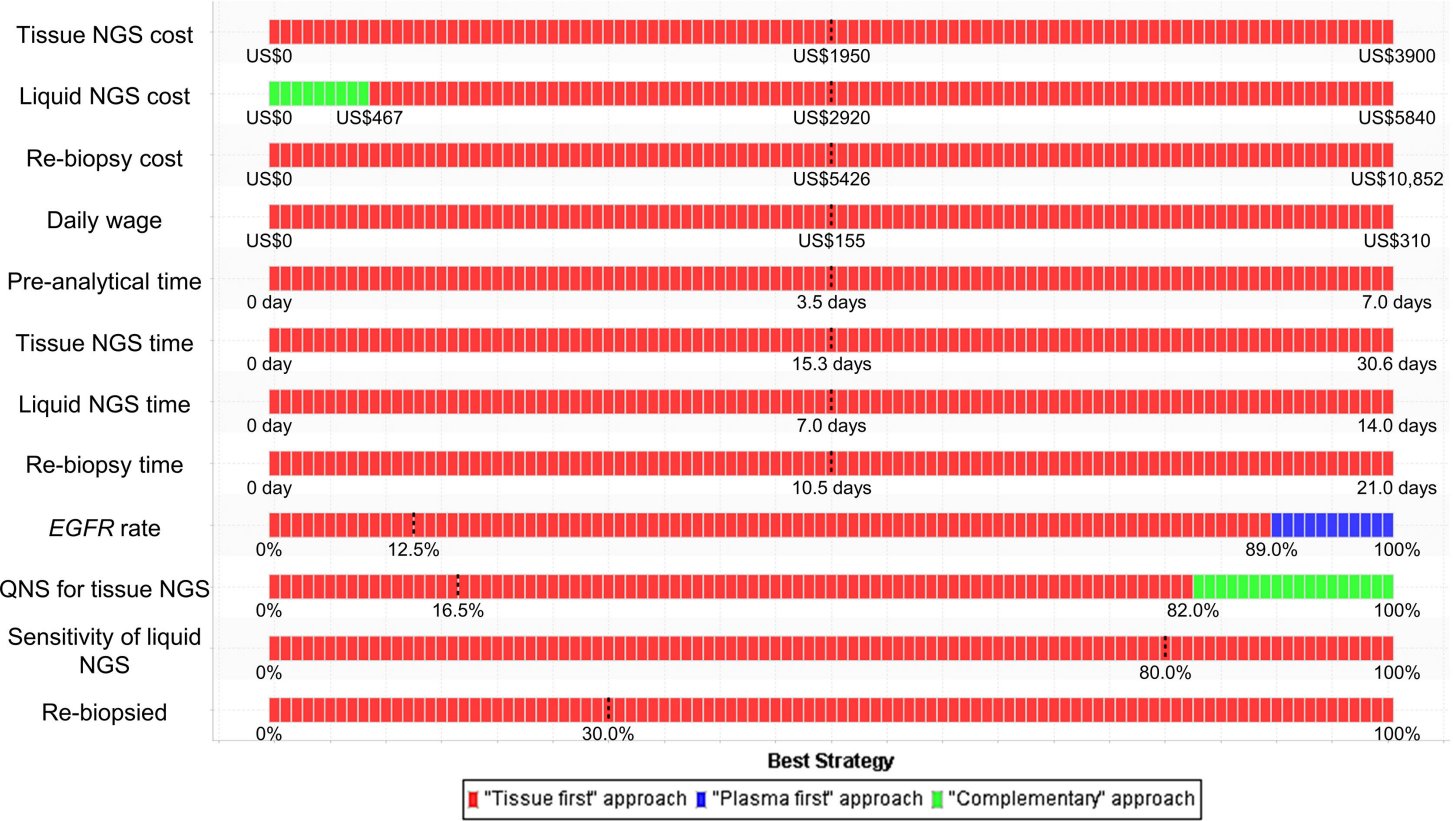
Stacked one-way sensitivity analysis for minimizing monetary loss. We performed a series of one-way sensitivity analyses by varying parameter values in broad ranges (**Table 1**) and determined the best strategy at each value. The dashed lines represent the baseline values. *EGFR*, epidermal growth factor receptor; NGS, next-generation sequencing; QNS, quantity not sufficient.

Stacked one-way sensitivity analyses showed that the major determinants in minimizing monetary loss were the cost of liquid-based NGS, prevalence rate of the *EGFR* mutation, and probability of specimens insufficient for tissue-based NGS (**Figure 2**). When the values of other parameters were not changed, the complementary NGS approach would be the best strategy when the cost of liquid-based NGS was reduced to US$467 or the probability of specimens insufficient for the tissue-based NGS was increased to 82.0%. If the prevalence rate of the *EGFR* mutation was higher than 89.0%, the plasma-first NGS approach would be the best strategy in minimizing monetary loss. The tissue-first NGS approach remained the best strategy if the other parameters varied in broad ranges.

**Figure 3:**
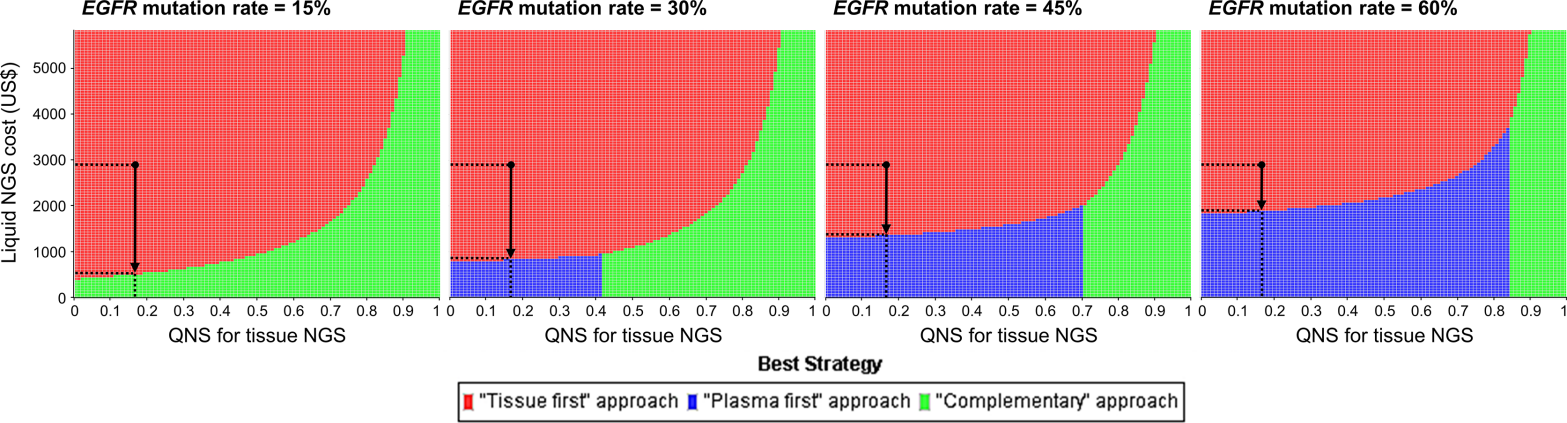
Three-way sensitivity analysis for minimizing monetary loss. The black dots represent the baseline cost of liquid-based NGS and the probability that specimens are insufficient for tissue-based NGS. In a population whose *EGFR* mutation rate was 15%, complimentary NGS approach would be preferable if the price of liquid-based NGS was reduced to US$526; and plasma-first NGS approach would become preferable if its price in USD was reduced to $818, $1,343, and $1,869 given the *EGFR* mutation rate of 30%, 45%, and 60% respectively (vertical arrows). *EGFR*, epidermal growth factor receptor; NGS, next-generation sequencing; QNS, quantity not sufficient.

Three-way sensitivity analysis revealed that if the cost of the liquid-based NGS decreased or the probability of specimens insufficient for the tissue-based NGS increased, the complementary or plasma-first NGS approach would be the best strategy (**Figure 3**). For example, given a population whose *EGFR* mutation rate was 15%, the complementary NGS approach would become a preferable strategy if the price of liquid-based NGS could be reduced to US$526. The plasma-first NGS approach, however, would be the best strategy if its testing price, in USD, was reduced to $818, $1,343, and $1,869 for populations with *EGFR* mutation rates of 30%, 45%, and 60%, respectively. If there is a high probability of having insufficient specimens for tissue-based NGS, the complementary NGS approach would be the best strategy.

When compared with the base-case results, the costs and monetary losses in sensitivity analysis using a DNA panel were lower (**Supplementary Table 2**). However, lower percentages of patients with appropriate FDA-approved therapies were also identified. The tissue-first NGS approach remained the most cost-efficient strategy and the complementary NGS was the least time-consuming.

### Scenario Analysis Using Taiwanese Data

Results of scenario analysis using Taiwanese data are shown in **Supplementary Table 3**. The tissue-first NGS approach was the most cost-efficient strategy and the complementary NGS approach was the least time-consuming strategy. Nevertheless, the plasma-first NGS approach was better than complementary NGS approach in terms of minimizing monetary loss. The tissue-first NGS approach was more likely to be the best strategy in Taiwan given the varying parameter values in broad ranges (**Supplementary Figure 2**). Even if the *EGFR* mutation rate was 15%, the plasma-first NGS approach was the second alternative if the price of liquid-based NGS could be reduced (**Supplementary Figure 3**).

## Discussion

Although previous studies have demonstrated that the prevailing tissue-based NGS is a cost-efficient and time-saving strategy (5) and liquid-based NGS for patients, with insufficient tissue specimens, adds lives with a modest budget impact (8), there has been no research comparing the testing costs and testing turnaround times of three NGS approaches. This study addressed the false-negative results of liquid-based NGS and re-biopsy issues. We conducted a wide literature search for gene alterations rates and testing turnaround times. By integrating testing and time costs to calculate and compare the monetary losses of the three approaches, we found that the tissue-first NGS approach was the best testing strategy (**Table 2**). The complementary NGS approach was the alternative option for a population with a low prevalence rate of *EGFR* mutation, whereas the plasma-first NGS approach would become increasingly preferable as the *EGFR* mutation rates increase (**Figure 3**). These results could help the health administrators plan their reimbursement policies pertaining to NGS testing.

Contrary to a previous investigation, which showed that determining the biomarker status of lung cancer *via* blood-based testing was less expensive than *via* tissue-based testing due to fewer complications (31), we found that the plasma-first NGS approach incurred more costs and was more time-consuming than the tissue-first NGS approach, leading to a greater monetary loss. A possible explanation for the contradicting results was that we considered a tissue-based NGS after a negative liquid-based NGS, whereas the aforementioned investigators did not. Subsequent tissue-based NGS not only increased the testing costs but also extended the testing turnaround times and it added a possibility of re-biopsy. Since the cost for procedure-related complications has already been included in the cost of re-biopsy [**Table 1** (10, 11)], we believe that our study results are valid.

The cost for liquid-based NGS, the prevalence rate of *EGFR* mutation, and the quantity of tissue specimens are major determinants in minimizing monetary loss (**Figure 2**). The best NGS approach will depend on the interaction of these factors. For example, given a population whose *EGFR* mutation is 15%, the complementary NGS approach would be preferable if the price of liquid-based NGS is lowered to US$526 (**Figure 3**). In contrast, in a population whose *EGFR* mutation rate is 45% or 60%, such as in non-smoking Caucasians or Asian Americans (32), the plasma-first NGS approach would become the best strategy given the reduced cost for liquid-based NGS. In Taiwan, where the price of liquid-based NGS remained high due to a small daily testing volume, tissue-first NGS approach would be the best strategy (**Supplementary Figure 3**). However, because the average daily wage (**Supplementary Table 1**) is much lower than that in the U.S., the effect of time costs on monetary loss would become less obvious. Consequently, the time-consuming plasma-first NGS approach turned out to be the alternative even if the *EGFR* mutation rate was 15%.

We did not consider additional cost and time related to testing for PD-L1 expression levels, which should be performed when there were no actionable gene alterations. Since the percentages of actionable gene alterations detected by three NGS approaches were similar, testing for PD-L1 expression levels should not confound the results. Leighl et al. reported that when using the plasma-first NGS approach, tissue specimens may be saved for the future testing of PD-L1 expression levels (17). However, liquid-based NGS has a false-negative rate of up to 30% (33) and patients with a negative result on liquid-based NGS will require further tumor tissue genotyping. We believe that the structure of our decision tree model is reasonable. Conversely, we did not regard tumor mutation burden as an actionable gene alteration. Although there are several promising investigations (34, 35), guidelines have not yet recommended its routine use in clinical practice (2).

There were several limitations in our study. First, we hypothesized that patients entered into the model with tissue samples available for tumor genotyping. In fact, for those with tissue samples unavailable for tumor genotyping, the plasma-first approach might act as the best choice. However, sensitivity analyses for the probability of insufficient tissue specimens were performed. When the probability of obtaining specimens insufficient for tissue-based NGS was 100%, the best strategy was complementary instead of the plasma-first NGS approach. Second, though we had considered the time cost, the transportation and caregiver costs were not estimated in this study. As a result, costs rendered on each strategy might have been underestimated. While calculating the time cost, we also did not account the time spent in decision making and delayed treatment, which further underestimated the time cost. Third, we only compared the testing costs and testing turnaround times, the cost and effectiveness after each testing strategy were not evaluated. Nevertheless, as long as the percentages of patients with appropriate FDA-approved therapies were comparable across three NGS approaches, the costs and effectiveness of three testing strategies would be similar.

In conclusion, the tissue-first NGS approach is currently the best strategy in minimizing monetary loss. The complementary NGS approach is an alternative for populations with a low prevalence rate of EGFR mutation, and the plasma-first NGS approach would become increasingly preferable as the *EGFR* mutation rates increase.

## Data Availability Statement

The original contributions presented in the study are included in the article/**Supplementary Material**. Further inquiries can be directed to the corresponding author.

## Author Contributions

S-CY had full access to all the data in the study and takes responsibility for data integrity and the accuracy of the data analysis. Study concept and design: S-CY and W-CS. Acquisition, analysis, or interpretation of data: S-CY and C-CL. Drafting of the manuscript: S-CY. Critical revision of themanuscript for important intellectual content: All authors. Statistical analysis: S-CY. Obtained funding: S-CY. Administrative, technical, or material support: YLC. Supervision: W-CS. All authors contributed to the article and approved the submitted version.

## Funding

This work was supported by the Ministry of Science and Technology (110-2314-B-006-100-MY2) and National Cheng Kung University Hospital (NCKUH-11102033). The funder had no role in the design and conduct of the study; collection, management, analysis, and interpretation of the data; preparation, review, or approval of the manuscript; and decision to submit the manuscript for publication.

## Conflict of Interest

S-CY reports grants from the Ministry of Science and Technology and National Cheng Kung University Hospital during the conduct of the study.

The remaining authors declare that the research was conducted in the absence of any commercial or financial relationships that could be construed as a potential conflict of interest.

## Publisher’s Note

All claims expressed in this article are solely those of the authors and do not necessarily represent those of their affiliated organizations, or those of the publisher, the editors and the reviewers. Any product that may be evaluated in this article, or claim that may be made by its manufacturer, is not guaranteed or endorsed by the publisher.
