# Supplementary material for: Economic Analysis of Tissue-First, Plasma-First, and Complementary NGS Approaches for Treatment-Naïve Metastatic Lung Adenocarcinoma
Source: Front Oncol. 2022 May 20;12:873111. doi: 10.3389/fonc.2022.873111 (PMC9163561; doi:10.3389/fonc.2022.873111)
Supplement: Supplementary file 1 [file DataSheet_1.docx]

Supplementary material

**Economic analysis of tissue-first, plasma-first, and complementary NGS approaches for treatment-naïve metastatic lung adenocarcinoma**

**Supplementary Figure 1** Decision tree analysis using Taiwanese data. ^a^Including cost for liquid-based NGS. ^b^Monetary loss included both testing and productivity costs, latter was the product of turnaround time and average wage (**Supplementary** **Table 1**). FDA, U.S. Food and Drug Administration. NGS, next-generation sequencing; QNS, quantity not sufficient.


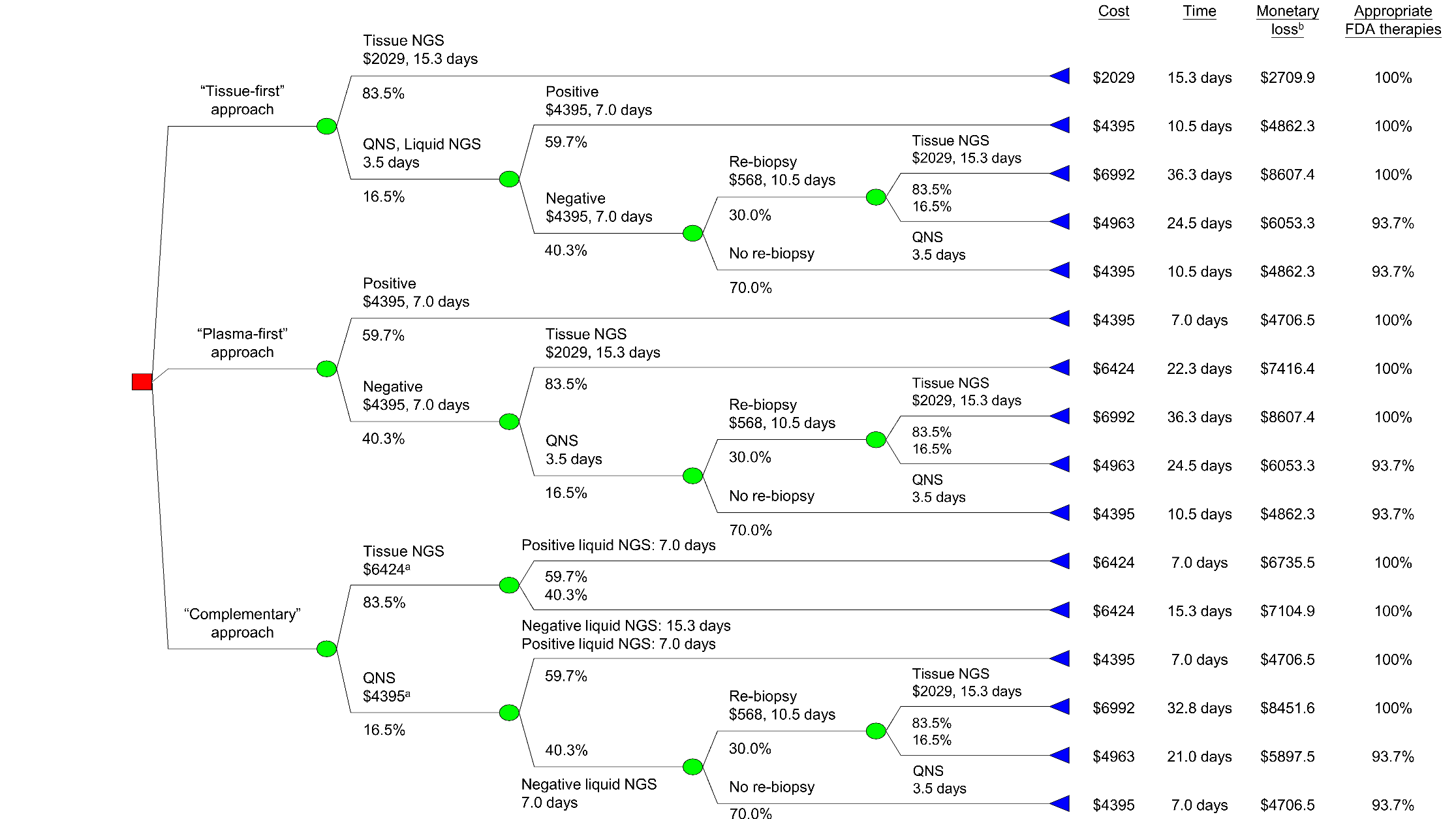


**Supplementary Figure 2** Stacked one-way sensitivity analysis using Taiwanese data. The dashed lines represent the baseline values. *EGFR*, epidermal growth factor receptor; NGS, next-generation sequencing; QNS, quantity not sufficient.


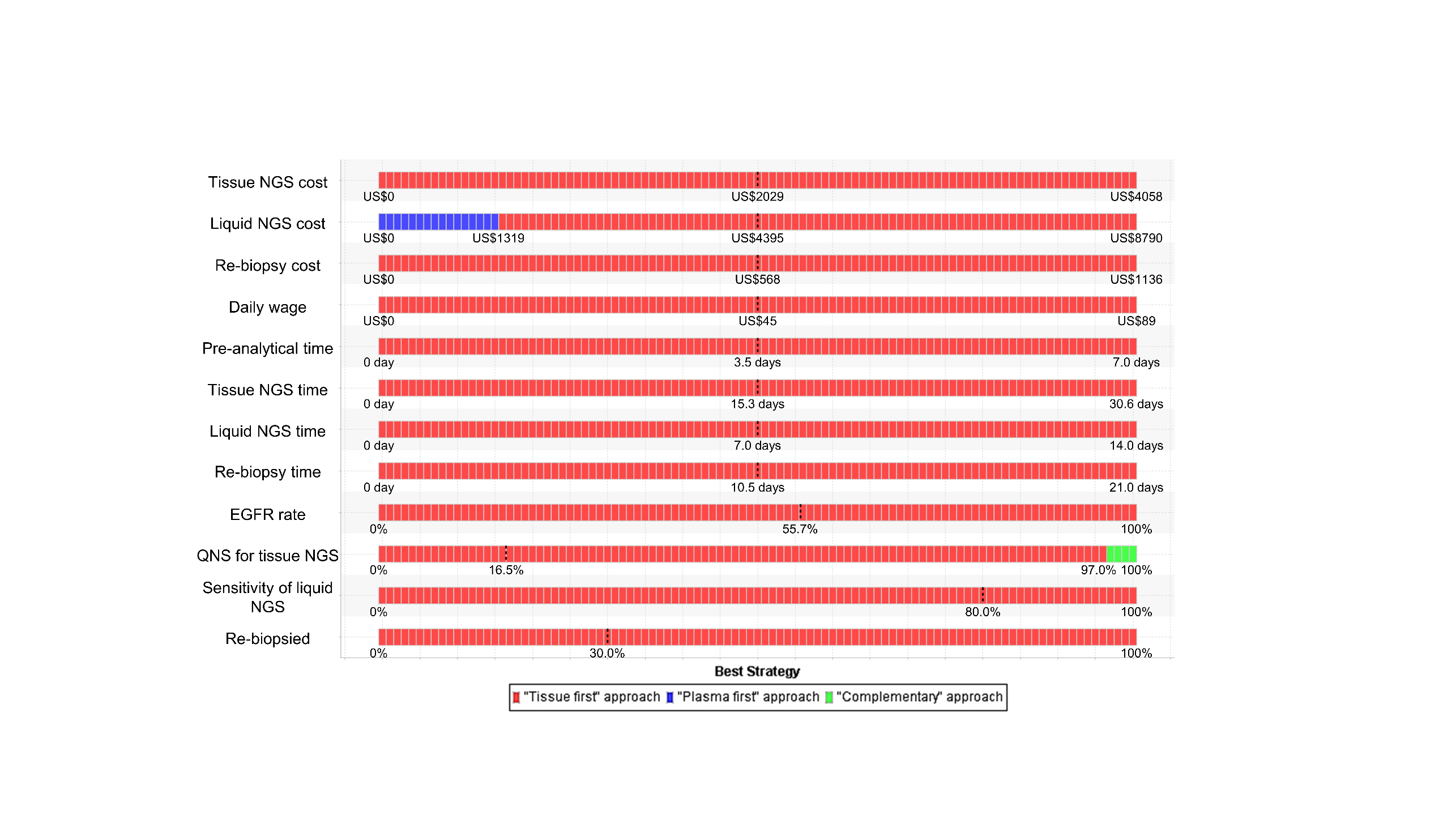


**Supplementary Figure 3** Three-way sensitivity analysis using Taiwanese data. The black dots represent the baseline cost of liquid-based NGS and the probability that specimens are insufficient for tissue-based NGS. Plasma-first NGS approach would be preferable if the price (USD) of liquid-based NGS was reduced to 440, 791, 1143, 1406 given the *EGFR* mutation rate of 15%, 30%, 45%, and 60% respectively (vertical arrows). *EGFR*, epidermal growth factor receptor; NGS, next-generation sequencing; QNS, quantity not sufficient.

**
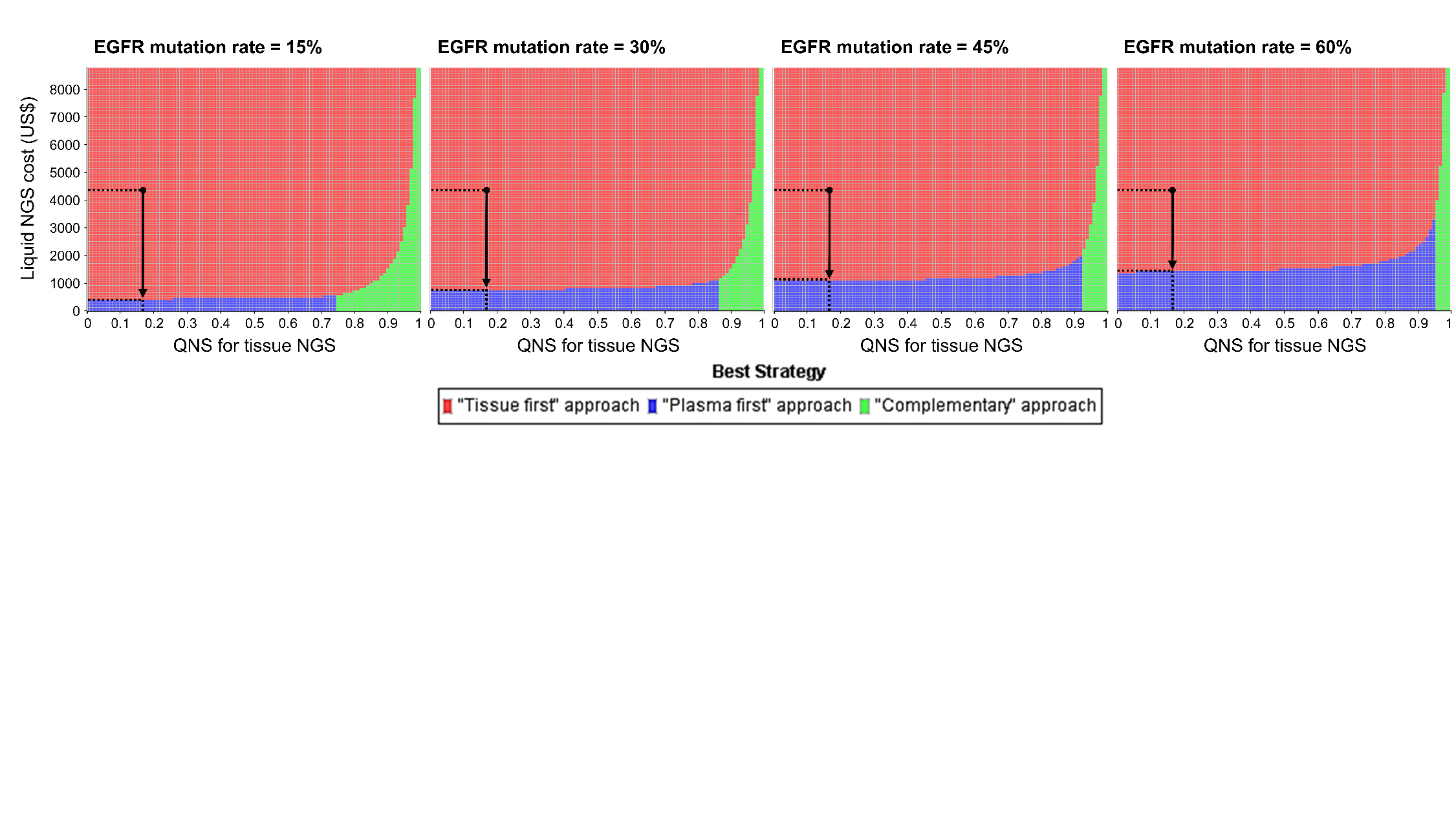
**

| **Supplementary Table 1** Input parameters using Taiwanese data | | | | | |
| --- | --- | --- | --- | --- | --- |
| Parameter | Baseline value | Range | | Distribution | References for baseline value |
|  |  | Minimum | Maximum |  |  |
| Testing cost (US$) | | | | | |
| Tissue-based NGS | 2029 | 0 | 4058 | Gamma (100,20.29) | expert survey |
| Liquid-based NGS | 4395 | 0 | 8790 | Gamma (100,43.95) | expert survey |
| Re-biopsy | 568 | 0 | 1136 | Gamma (100,5.68) | (1) |
| Average wage (US$/day) | 45 | 0 | 89 | Gamma (100,0.445) | (2) |
| Turnaround time (day) | | | | | |
| Pre-analytical^a^ | 3.5 | 0 | 7.0 | Gamma (100,0.035) | (3) |
| Tissue-based NGS | 15.3 | 0 | 30.6 | Gamma (100,0.153) | (3) |
| Liquid-based NGS | 7.0 | 0 | 14.0 | Gamma (100,0.07) | (4) |
| Re-biopsy | 10.5 | 0 | 21.0 | Gamma (100,0.105) | (5) |
| Gene alteration rate | | | | | |
| *EGFR* | 55.7% | 0% | 100% | Beta (987,785) | (6) |
| *ALK* | 4.2% |  |  | Beta (13,299) | (7) |
| *ROS1* | 2.4% |  |  | Beta (12,480) | (8) |
| *BRAF V600E* | 0.7% |  |  | Beta (12,1760) | (6) |
| *RET* | 1.4% |  |  | Beta (2,140) | (9) |
| *METex14* | 4.0% |  |  | Beta (27,641) | (10) |
| *NTRK* | 0.2% |  |  | Beta (1,540) | (11) |
| *KRAS G12C* | 1.5% |  |  | Beta (81,5197) | (12) |
| *HER2* | 4.5% |  |  | Beta (40,848) | (13) |
| Re-biopsy input | | | | | |
| Sensitivity of liquid-based NGS^b^ | 80.0% | 0% | 100% | Beta (48,12) | (14) |
| Quantity not sufficient (QNS) for tissue-based NGS | 16.5% | 0% | 100% | Beta (14,71) | (15) |
| Patients re-biopsied of those in need | 30.0% | 0% | 100% | Beta (30,70) | (5) |
| Patients with appropriate FDA-approved therapies using tissue-based NGS | 100% |  |  | -- | -- |
| Patients with appropriate FDA-approved therapies using liquid-based NGS^c^ | 93.7% |  |  | Beta (177,12) | (14) |
| ^a^ Pre-analytical time of tissue-based NGS. The turnaround time of tissue-based NGS includes both the pre-analytical and in-laboratory time.  ^b^ Negative liquid-based NGS (%) = 100% - (*EGFR/ALK/ROS1/BRAF V600E/RET/METex14/NTRK/KRAS G12C/HER2* alteration rate(6-13): 74.7% × sensitivity of liquid-based NGS(14): 80%) = 40.3%.  ^c^ 100% - false-negative rate (%) of liquid-based NGS.  *ALK*, anaplastic lymphoma kinase; *BRAF*, *B-Raf* proto-oncogene; *EGFR*, epidermal growth factor receptor; FDA, U.S. Food and Drug Administration; *HER2*, human epidermal growth factor receptor 2; *METex14*, mesenchymal-epithelial transition exon 14; NGS, next generation sequencing; *NTRK*, neurotrophic tyrosine receptor kinase; *KRAS*, *K-Ras* proto-oncogene; *RET*, rearranged during transfection; *ROS1*, ROS proto-oncogene 1. | | | | | |

**References:**

1. Yang SC, Yeh YC, Chen YL, Chiu CH. Economic analysis of exclusionary EGFR test versus upfront NGS for lung adenocarcinoma in high EGFR mutation prevalence areas. *J Natl Compr Cancer Netw* (2022) 6:1-9. doi: 10.6004/jnccn.2021.7120.

2. National Statistics, Taiwan. *Monthly Income of Major Job for Employees.* Available at: https://www.stat.gov.tw/ct.asp?xItem=46590&ctNode=3579&mp=4 (Accessed 4 January 2022).

3. Dagogo-Jack I, Robinson H, Mino-Kenudson M, Farago AF, Kamesan V, Iafrate AJ, et al. Expediting comprehensive molecular analysis to optimize initial treatment of lung cancer patients with minimal smoking history. *J Thorac Oncol* (2019) 14(5):835-43. doi: 10.1016/j.jtho.2018.12.032.

4. Guardant360^®^ CDx. *How do you treat at the speed of cancer? The answers are in our blood.* Available at: https://guardant360cdx.com (Accessed 1 November 2021).

5. Pennell NA, Mutebi A, Zhou ZY, Ricculli ML, Tang W, Wang H, et al. Economic impact of next-generation sequencing versus single-gene testing to detect genomic alterations in metastatic non-small-cell lung cancer using a decision analytic model. *JCO Precis Oncol* (2019) 3:1-9. doi: 10.1200/po.18.00356.

6. Hsu KH, Ho CC, Hsia TC, Tseng JS, Su KY, Wu MF, et al. Identification of five driver gene mutations in patients with treatment-naive lung adenocarcinoma in Taiwan. *PLoS One* (2015) 10:e0120852. doi: 10.1371/journal.pone.0120852.

7. Wu YC, Chang IC, Wang CL, Chen TD, Chen YT, Liu HP, et al. Comparison of IHC, FISH and RT-PCR methods for detection of ALK rearrangements in 312 non-small cell lung cancer patients in Taiwan. *PLoS One* (2013) 8:e70839. doi: 10.1371/journal.pone.0070839.

8. Chen YF, Hsieh MS, Wu SG, Chang YL, Shih JY, Liu YN, et al. Clinical and the prognostic characteristics of lung adenocarcinoma patients with ROS1 fusion in comparison with other driver mutations in East Asian populations. *J Thorac Oncol* (2014) 9:1171-9. doi: 10.1097/jto.0000000000000232.

9. Wu SG, Liu YN, Yu CJ, Yang JC, Shih JY. Driver mutations of young lung adenocarcinoma patients with malignant pleural effusion. *Genes Chromosomes Cancer* (2018) 57:513-21. doi: 10.1002/gcc.22647.

10. Gow CH, Hsieh MS, Wu SG, Shih JY. A comprehensive analysis of clinical outcomes in lung cancer patients harboring a MET exon 14 skipping mutation compared to other driver mutations in an East Asian population. *Lung Cancer* (2017) 103:82-9. doi: 10.1016/j.lungcan.2016.12.001.

11. Okamura R, Boichard A, Kato S, Sicklick JK, Bazhenova L, Kurzrock R. Analysis of NTRK alterations in pan-cancer adult and pediatric malignancies: implications for NTRK-targeted therapeutics. *JCO Precis Oncol* (2018) 2018:PO.18.00183. doi: 10.1200/po.18.00183.

12. Wu SG, Liao WY, Su KY, Yu SL, Huang YL, Yu CJ, et al. Prognostic characteristics and immunotherapy response of patients with nonsquamous NSCLC with KRAS mutation in East Asian populations: a single-center cohort study in Taiwan. *JTO Clinical and Research Reports* (2021) 2:100140. doi: 10.1016/j.jtocrr.2020.100140.

13. Gow CH, Chang HT, Lim CK, Liu CY, Chen JS, Shih JY. Comparable clinical outcomes in patients with HER2-mutant and EGFR-mutant lung adenocarcinomas. *Genes Chromosomes Cancer* (2017) 56:373-81. doi: 10.1002/gcc.22442.

14. Leighl NB, Page RD, Raymond VM, Daniel DB, Divers SG, Reckamp KL, et al. Clinical utility of comprehensive cell-free DNA analysis to identify genomic biomarkers in patients with newly diagnosed metastatic non-small cell lung cancer. *Clin Cancer Res* (2019) 25(15):4691-700. doi: 10.1158/1078-0432.CCR-19-0624.

15. Goswami RS, Luthra R, Singh RR, Patel KP, Routbort MJ, Aldape KD, et al. Identification of factors affecting the success of next-generation sequencing testing in solid tumors. *Am J Clin Pathol* (2016) 145(2):222-37. doi: 10.1093/ajcp/aqv023.

| **Supplementary Table 2** Sensitivity analysis using a deoxyribonucleic acid (DNA) panel of tissue-based NGS^a^ | | | | |
| --- | --- | --- | --- | --- |
|  | Cost (US$) | Time (day) | Monetary loss^b^ (US$) | Patients with appropriate FDA-approved therapies |
| “Tissue-first” NGS approach | 1203 (911 to 1576) | 15.4 (13.0 to 18.1) | 3607 (2938 to 4341) | 91.5% (86.5 to 95.1%) |
| “Complementary” NGS approach | 3644 (3060 to 4237) | 13.3 (11.2 to 15.6) | 5716 (4949 to 6443) | 91.5% (86.5 to 95.1%) |
| “Plasma-first” NGS approach | 3532 (2957 to 4137) | 18.3 (15.7 to 21.1) | 6381 (5506 to 7244) | 91.5% (86.5 to 95.1%) |
| ^a^ We applied a unit cost of US$598 (CPT: 81445) for the DNA panel of tissue-based NGS. Values in parentheses denote the 95% prediction intervals.  ^b^ Monetary loss included testing and productivity costs, the latter was the product of turnaround time and average wage.  CPT, Current Procedural Terminology code; FDA, U.S. Food and Drug Administration; NGS, next generation sequencing. | | | | |

| **Supplementary Table 3** Scenario analysis using Taiwanese data^a^ | | | | |
| --- | --- | --- | --- | --- |
|  | Cost (US$) | Time (day) | Monetary loss^b^ (US$) | Patients with appropriate FDA-approved therapies |
| “Tissue-first” NGS approach | 2462 (2047 to 2889) | 15.0 (12.6 to 17.5) | 3133 (2690 to 3590) | 99.7% (99.4 to 99.9%) |
| “Plasma-first” NGS approach | 5118 (4231 to 5990) | 12.8 (10.8 to 15.1) | 5690 (4794 to 6574) | 99.7% (99.4 to 99.9%) |
| “Complementary” NGS approach | 6137 (5175 to 7063) | 10.2 (8.8 to 11.8) | 6595 (5635 to 7510) | 99.7% (99.4 to 99.9%) |
| ^a^ Values in parentheses denote the 95% prediction intervals.  ^b^ Monetary loss included testing and productivity costs, latter was the product of turnaround time and average wage.  FDA, U.S. Food and Drug Administration; NGS, next generation sequencing. | | | | |
